# Supplementary material for: Phase stabilization by electronic entropy in plutonium
Source: Nat Commun. 2019 Jul 18;10:3159. doi: 10.1038/s41467-019-11166-0 (PMC6639308; doi:10.1038/s41467-019-11166-0)
Supplement: Supplementary file 1 — Supplementary Information [file 41467_2019_11166_MOESM1_ESM.pdf]

Phase stabilization by electronic entropy in plutonium, Harrison *et al.*, Supplementary Notes, Tables and Figures.

Supplementary Note 1

Comparison to the Invar model thermodynamic treatment: Magnetostriction measurements are necessary for accurately separating the electronic and phonon contributions to the lattice, causing prior efforts to separate these contributions in the absence of magnetostriction data to be of limited success.[1] Because the upper excitation energy E_2^* leads to a negative thermal expansion, which is obviously quite distinct from the Debye function, this higher excitation energy was successfully extracted in prior studies. However, because the positive thermal expansion associated with E_1^* has the same sign as the thermal expansion caused by phonons, it was subsequently missed. In contrast to our three level model, the two level Invar model was shown to be unable to account for the excess entropy and heat capacity measured in δ-Pu.[1]

Significant changes to the free energy partition function in the present approach include (i) the addition of effective magnetic moments associated with each configuration, (ii) the addition of a third level, which is necessary for accurately reproducing the forms of the magnetostriction and heat capacity, and (iii) a more realistic modeling of the volume-dependences of the various configurations, which are assumed to have well defined minima in accordance with electronic structure calculations.[2,3] The latter approach enables the bulk modulus to be derived from the partition function, which was not possible in in the Invar model without arbitrarily adding an extra term to the free energy.[1]

An underlying weakness of the Invar model,[1] is that atomic sites with different configurations were assumed to have different volumes, for which there is no evidence in δ-$Pu_{1-x}Ga_x$.[4]

In the present approach, by contrast, the volume V is the same for each of the configuration. Only the dependence of E_i on volume are assumed to be different.

Supplementary Note 2

Expanding fitting to include the magnetic susceptibility: The obtained values of E_1^* and E_2^* continue to be robust when the model to which the data is fit is expanded to include other quantities, such as the magnetic susceptibility. While the magnetostriction and thermal expansion measurements indicate that Ga-stabilized δ-Pu most likely settle into a non-magnetic or weakly magnetic configuration at low temperatures, heat capacity and magnetic susceptibility measurements indicate the coexistence of a Fermi liquid state, in which both the Sommerfeld coefficient[5] and Pauli susceptibility[6] are enhanced. In the mixed level picture, in which the ground state configuration consists of $n_f = 4$ $5f$-electrons confined to the atomic core, the Fermi liquid state is predicted to originate from the one $5f$-electron that is itinerant, and its hybridization with other states.[7] If the ground state configuration consists, instead, of that with $n_f = 5$ $5f$-electrons confined to the atomic core, then a Fermi liquid state could result from their weak hybridization with other states.

Any attempt to model the magnetic susceptibility requires additional fitting parameters to be introduced and is ultimately limited by the relative scarcity of available experimental data as a function of both T and x.[6,8] One approach is to utilize the methodology in which a finite $T_{\rm fl}$ mimics the behavior of a Fermi liquid at low temperatures.[9,10] In this case, both $T_{\rm fl}$ and the magnetic moment of the ground state configuration, μ_0^*, must acquire finite values. In order to reconcile the magnitude of the magnetic moment inferred from longitudinal magnetostriction and magnetic susceptibility measurements, η also needs to be considered as an adjustable

parameter. The magnetic susceptibility is given by $\chi_{zz} = \mu_0 \partial M_{zz}/\partial B$, where

$$M_{zz}(T,B) = -\frac{\partial F_{\rm el}}{\partial B} = \frac{1}{Z_{\rm el}{}^*} \sum_{i=0,1,2} 2\mu_i^* {\rm e}^{-\frac{k_{\rm B}E_i^*}{k_{\rm B}T'}} \sinh\left(\frac{\mu_i^* B}{k_{\rm B}T'}\right). \tag{1}$$

The results of a combined fit to the magnetostriction, volume expansion and magnetic susceptibility are shown in Supplementary Table 1 and Supplementary Fig. 4.

An alternative approach is to substitute the $\sinh(\mu_i^* B/k_{\rm B}T')$ term for the ground state configuration in Supplementary Equation 1 with a Fermi gas-like form

$$\mathrm{f}(E,B,W,\mu_{\rm F}^*) = \frac{2}{\sqrt{\pi}W} \int_{-\infty}^{\infty} \sum_{\sigma=\pm\frac{1}{2}} 2\sigma {\rm e}^{-\left(\frac{E+2\sigma\mu_{\rm F}^* B}{W}\right)^2} \mathrm{f}_{\rm FD}(E,T) {\rm d}E, \tag{2}$$

where we have assumed a Gaussian line shape for the electronic density-of-states, given the unknown band topology. Here, $\mathrm{f}_{\rm FD} = (1+e^{\frac{E}{T}})^{-1}$ is the Fermi-Dirac distribution function while W is the electronic bandwidth. The results of a combined fit to the magnetostriction, volume expansion and magnetic susceptibility are shown in Supplementary Table 2 and Supplementary Fig. 4.

While the former approach using $T_{\rm fl} \neq 0$ yields small magnetic moments for the ground state configuration, the latter approach in which the ground state configuration is accompanied by a half-filled electronic band more accurately reproduces the form of the magnetic susceptibility for Ga-stabilized δ-Pu with $x = 6\%$ as a function of temperature.[8] With both approaches, the respective energy levels E_1^* and E_2^* for $x = 2\%$ and 7% change very little on including the magnetic susceptibility. A general prediction of both approaches is that the temperature-dependence of the magnetic susceptibility becomes stronger on reducing x, which is not too surprising given that volume magnetostriction is proportional to the volume-dependence of the magnetization. The development of a more refined model will require comprehensive measurements of the susceptibility as a function of both T and x.

Quantity	$x = 2\%$ Ga	$x = 7\%$ Ga	all x	Units
μ_0^*	0.0 ± 0.1	0.5 ± 0.2		μ_B
$(1+\nu_1^*)V_0$	24.64 ± 0.02	24.26 ± 0.11		Å^3
E_1^*	275 ± 10	458 ± 18		K
μ_1^*	1.4 ± 0.3	1.0 ± 0.3		μ_B
$(1+\nu_2^*)V_0$	21.30 ± 0.01	23.63 ± 0.08		Å^3
E_2^*	1360 ± 90	890 ± 80		K
μ_2^*	1.9 ± 1.0	2.5 ± 1.4		μ_B
T_{fl}	12 ± 50	58 ± 50		K
γ			0.54 ± 0.04	–
η	1.6 ± 0.4	1.4 ± 0.4		–

Supplementary Table 1: Values of the various parameters obtained on performing a least squares fit including the susceptibility with errors (estimated from their covariance). Again, $E_0^* = 0$.

Quantity	$x = 2\%$ Ga	$x = 7\%$ Ga	all x	Units
$(1+\nu_1^*)V_0$	24.64 ± 0.02	24.21 ± 0.11		Å^3
E_1^*	271 ± 10	388 ± 18		K
μ_1^*	1.3 ± 0.3	0.8 ± 0.3		μ_B
$(1+\nu_2^*)V_0$	21.41 ± 0.01	23.53 ± 0.08		Å^3
E_2^*	1320 ± 90	970 ± 80		K
μ_2^*	1.7 ± 1.0	2.5 ± 1.4		μ_B
γ			0.55 ± 0.04	–
W			408 ± 20	K
μ_F^*			0.8 ± 0.2	μ_B
η	1.3 ± 0.4	1.8 ± 0.4		–

Supplementary Table 2: Values of the various parameters obtained on performing a least squares fit including the susceptibility with errors (estimated from their covariance). Again, $E_0^* = 0$, and we have also set $T_\text{fl} = 0$.

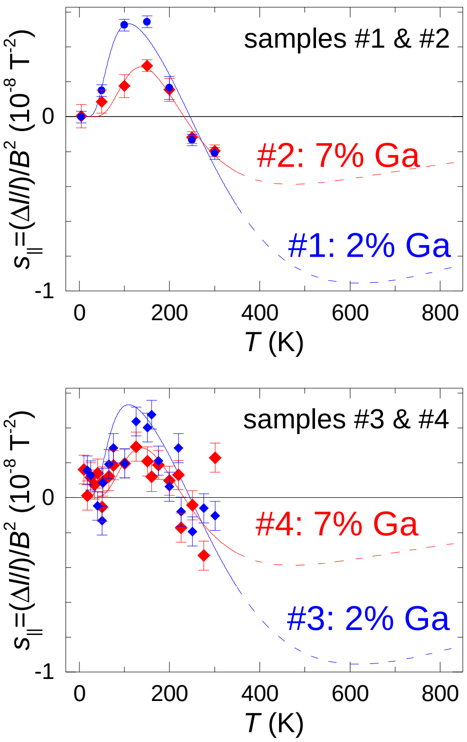

Supplementary Figure 1: Coefficients of the quadratic magnetostriction coefficient versus T for four different δ-Pu$_{1-x}$Ga$_x$ samples. a Measurements on samples #1 and #2 as plotted in Fig. 1c of the main paper, where blue circles refer to $x = 2\%$ and red diamonds refer to $x = 7\%$. The red and blue lines are the fits shown in Fig. 1c of the main text. **b** Measurements on samples #3 and #4, as referred to in the Methods section of the main paper, where blue diamonds refer to $x = 2\%$ and red diamonds refer to $x = 7\%$. In all cases, the error bars represent the standard error of the mean, estimated in each case from multiple sweeps at a single T.

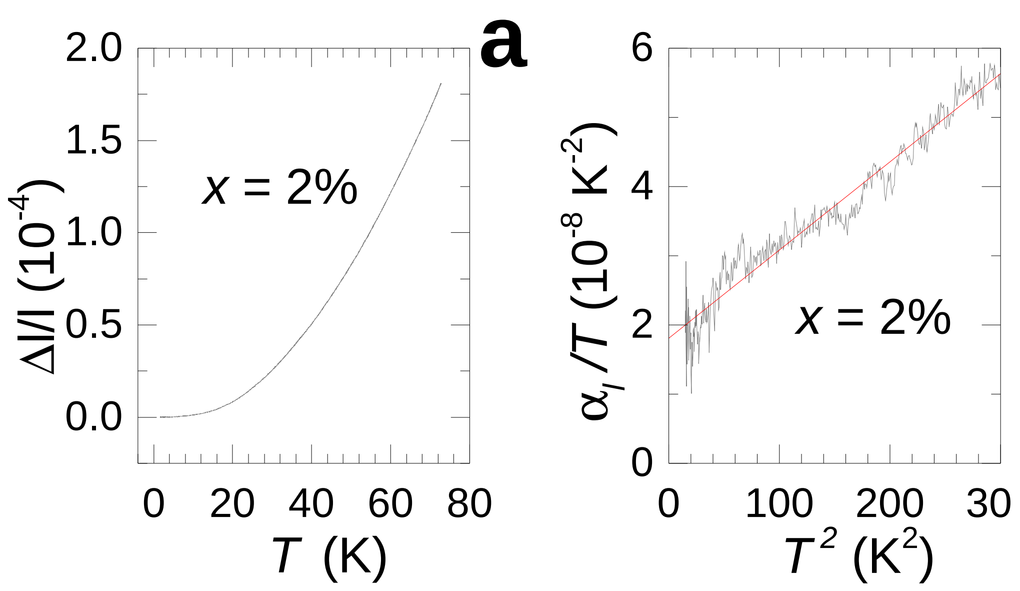

Supplementary Figure 2: Low temperature thermal expansion. **a** Change in length $\Delta l/l$ at low T in δ-$Pu_{1-x}Ga_x$ ($x = 2\%$) obtained using the optical fiber Bragg grating method. **b** Plot of α_l/T versus T^2 of δ-$Pu_{1-x}Ga_x$ ($x = 2\%$) at low T and $x = 2\%$ (grey curve), together with a linear fit (red line) to the function $\alpha_l/T = \frac{\kappa_0}{3}(k_{\rm ph}T^2 + \frac{2}{3}\gamma_{\rm el})$, where $k_{\rm ph}$ is a constant relating to phonons and $\gamma_{\rm el}$ is the linear-in-T electronic contribution to the heat capacity.[11] According to the fit, $\gamma_{\rm el} = (40 \pm 10)$ mJmol^{-1}K^{-2}, which is of comparable order to the result ($\gamma_{\rm el} = 64$ mJmol^{-1}K^{-2}) obtained for δ-$Pu_{1-x}Al_x$ ($x = 5\%$) from heat capacity measurements.[5]

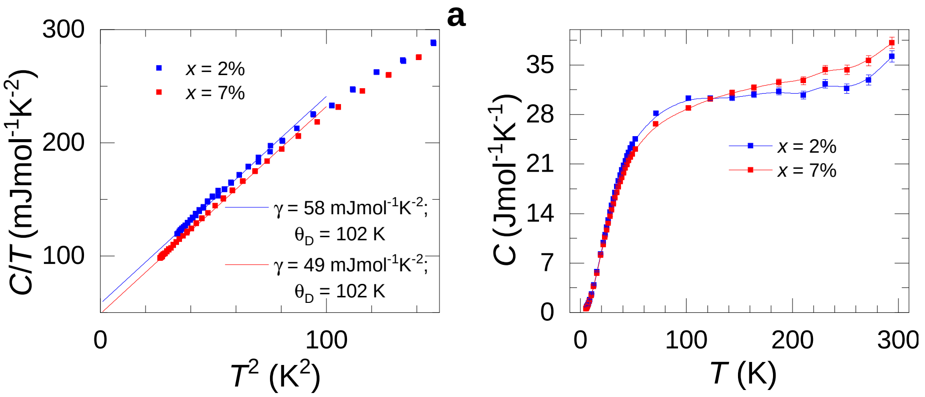

Supplementary Figure 3: Heat capacity for δ-Pu$_{1-x}$Ga$_x$. **a** C/T versus T^2 for samples #5 and #6 with $x = 2\%$ (blue squares) and $x = 7\%$ (red squares) Ga, respectively. The solid lines correspond to plots of the function $C/T = \gamma + (12\pi^4 RT^2)/(5\theta_{\rm D}^3)$,[5] where, here, γ refers to the Sommerfeld coefficient.[11] **b** C versus T for samples #5 and #6 with $x = 2\%$ and $x = 7\%$ Ga, respectively. Error bars indicate the standard error of the mean, while the lines are merely guides to the eye.

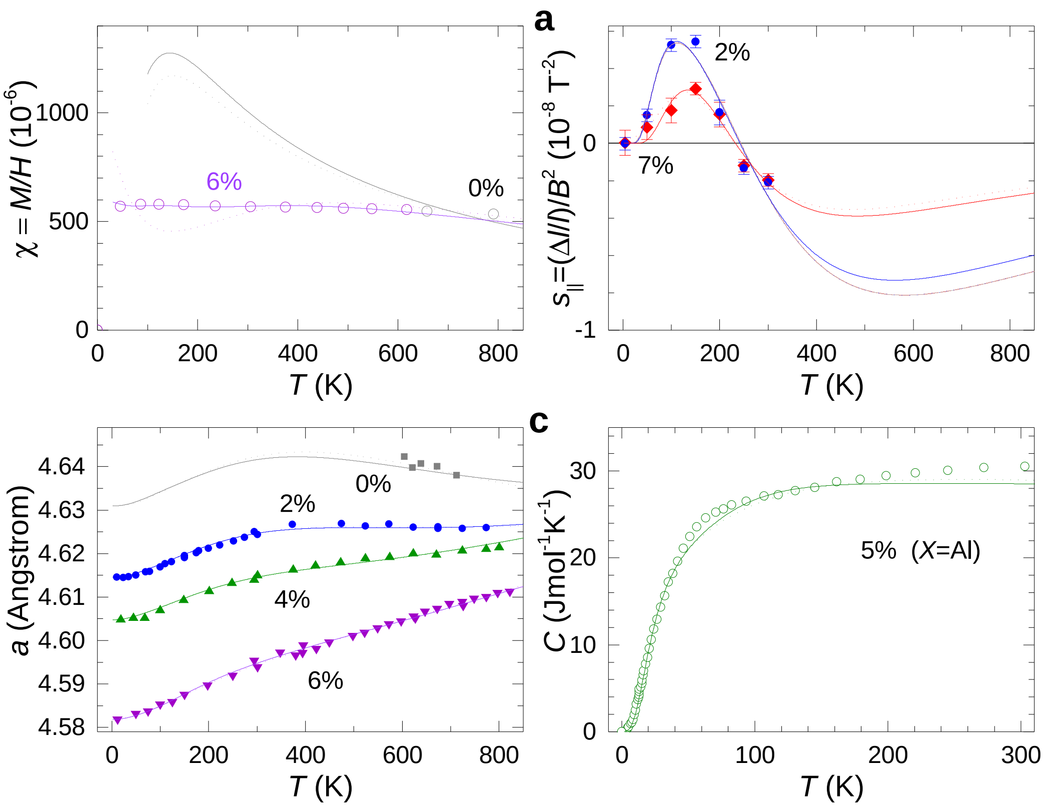

Supplementary Figure 4: Results of fitting expanded to include the susceptibility. **a** Magnetic susceptibility of δ-Pu$_{1-x}$Ga$_x$ versus T for $x = 0\%$ (light grey) and $x = 6\%$ (violet). **b** Magnetostriction coefficient of δ-Pu$_{1-x}$Ga$_x$ versus T for $x = 2\%$ (blue circles) and $x = 7\%$ (red diamonds). **c** Lattice parameter of δ-Pu$_{1-x}$Ga$_x$ versus T for $x = 0\%$ (light grey squares), 2% (blue circles), 4% (green up triangles) and $x = 6\%$ (violet down triangles). **d** Heat capacity of δ-Pu$_{1-x}$Al$_x$ versus T for $x = 5\%$ (open green circles). Data points represent experimental data referred to in the main text, while lines of the same color as the points refer to fits. The fits are made to the susceptibility, magnetostriction coefficient and lattice parameter using free energy functional either with T_{fl} included (dotted lines) as a finite parameter as contained in Supplementary Equation 1 or by using an alternative form given by Supplementary Equation 2 for the susceptibility (solid lines) that better reproduces a Fermi gas-like form for the ground state configuration (see Supplementary Note 2). No fits are made to the heat capacity. Instead, the lines are simply calculated from the free energy and compared against the measured data points.

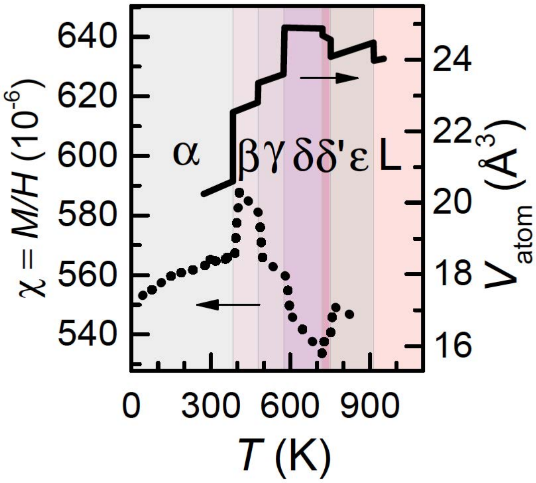

Supplementary Figure 5: Magnetovolume effect in Pu. Plots of the atomic volume (black line and right-hand-axis) and susceptibility (dotted line and left-hand-axis) of Pu versus temperature, with the different crystalline phases shaded in different colors for illustrative purposes.

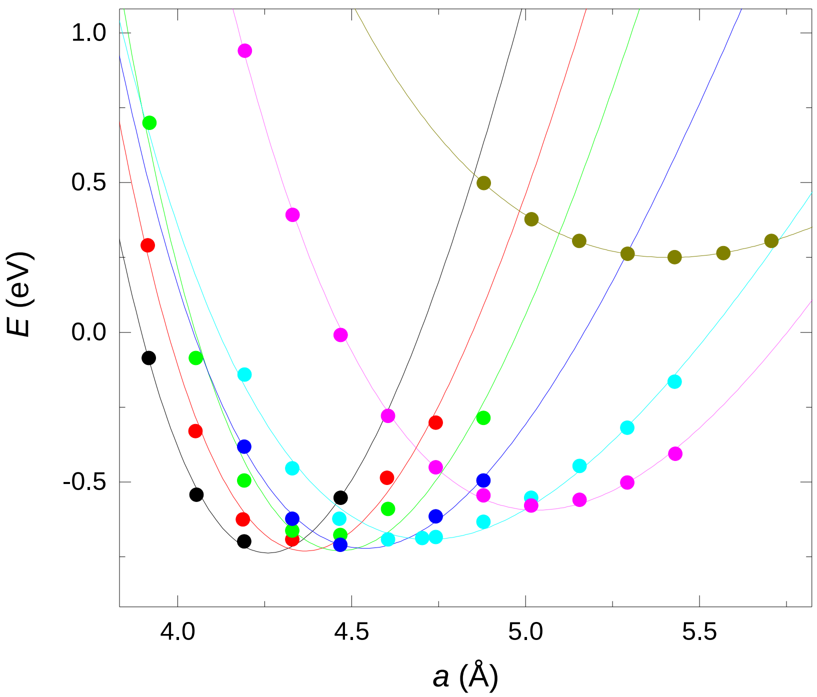

Supplementary Figure 6: Configurational energies versus volume according to Svane *et al.*[3]. Circle symbols are the energy E versus lattice parameter a calculated according to Svane *et al.*,[3] while lines are fits to Equation (9) in the Methods of the main paper. The colors of the circles and lines refer to different configurations with 0 (black), 1 (red), 2 (green), 3 (blue), 4 (cyan), 5 (magenta) and 6 (dark yellow) $5f$-electrons confined to the atomic core.

Supplementary References

[1] Lawson, A. C., Roberts, J. A., Martinez, B., Ramos, M., Kotliar, G., Trouw, F. W., Fitzsimmons, M. R., Hehlen, M. P., Lashley, J. C., Ledbetter, H., Mcqueeney, R. J., Migliori, A., Invar model for δ-phase Pu: thermal expansion, elastic and magnetic properties. *Phil. Mag.* **86**, 2713-2733 (2006).

[2] Eriksson, O., Becker, J. N., Balatsky, A. V., Wills, J. M., Novel electronic configuration in δ-Pu. *J. Alloy. & Comp.* **287**, 1-5 (1999).

[3] Svane, A., Petit, L., Szotek, Z., Temmerman, W. M., Self-interaction-corrected local spin density theory of $5f$-electron localization in actinides. *Phys. Rev. B* **76**, 115116 (2007).

[4] Migliori, A., Soderlind, P., Landa, A., Freibert, F. J., Maiorov, B., Ramshaw, B. J., Betts, J. B., Origin of the multiple configurations that drive the response of δ-plutonium's elastic moduli to temperature. *Proc. Nat. Acad. Sci. USA* **113**, 11158-11161 (2016).

[5] Lashley, J. C., Singleton, J., Migliori, A., Betts, J. B., Fisher, R. A., Smith, J. L., McQueeney, R. J. Experimental electronic heat capacities of α- and δ-plutonium: heavy fermion physics in an element. *Phys. Rev. Lett.* **91**, 205901 (2003).

[6] Lashley, J. C., Lawson, A., McQueeney, R. J., Lander, G. H., Absence of magnetic moments in plutonium. *Phys. Rev. B* **72**, 054416 (2005).

[7] Wills, J. W., Eriksson, O., Delin, A., Andersson, P. H., Joyce, J. J., Durakiewicz, T., Butterfield, M. T., Arko, A. J., Moore, D. P., Morales, L. A., A novel electronic configuration of the 5f states in δ-plutonium as revealed by the photo-electron spectra. *Journal of Electron Spectroscopy and Related Phenomena* **135**, 163-166 (2004).

[8] Méot-Reymond, S., Fourinier, J. M., Localization of $5f$ electrons in δ-plutonium: Evidence for the Kondo effect. *J. Alloys and Compounds* **232**, 119-125 (1996).

[9] Zieglowski, J., Häfner, H. U., Wohlleben, D. Volume magnetostriction of rare-earth metals with unstable $4f$ shells. *Phys. Rev. Lett.* **56**, 193-196 (1986).

[10] Wohlleben, D., *Physics and chemistry of electrons and ions in condensed matter* ed. Acrivos, J. V., Mott, N. F., Yoffe, A. D. (Reidel, Dordrecht 1984) pp. 85-107.

[11] Ashcroft, N. W., Mermin, N. D., *Solid state physics* (Saunders College Publishing, Orlando 1976).
